# The Cyclopædia of Practical Surgery

**Published:** 1834-07-01

**Authors:** 


*The Cyclopædia of Practical Surgery.* By WILLIAM BLOXAM, Esq., M.R.C.S. Revised by CHARLES MILLARD, Esq., M.R.C.S. Fasciculi I—IV.—London, 1834. Folio.

THE four fasciculi before us belong to Division I. "On the Surgical Anatomy of the Arteries." The first fasciculus contains a dissection of the arteries, veins, and nerves of the head and neck; the second, a dissection of the subclavian and axillary arteries; the third, the operative surgery of the carotid and subclavian arteries; and the fourth, the dissection of the arteries, veins, and nerves of the upper extremity. The drawings are made by Mr. Cane, curator to the museum of King's College, and are exceedingly well done. The plates are lithographic, and partly coloured, so as to give them the greatest possible distinctness.

We consider it our duty, as honest reviewers, again and again to caution our readers against buying books in fasciculi, numbers, or by whatever other name the useless fragments may be known. Wait at least till some great portion of the book is finished, which may form an instructive whole. In the present case the first portion will be completed in about twelve monthly parts, and we therefore counsel our readers to bridle their impatience, and buy Division I. in December.

